# Microstructure, Hardness and High-Temperature Corrosion Behaviors in Sulfur-Containing Environment of Laser Cladding Y_2_O_3_/IN625 Composite Coating

**DOI:** 10.3390/ma17194837

**Published:** 2024-09-30

**Authors:** Yong Li, Hao Zheng, Zhe Chang, Fuguang Liu, Yansong Wang, Yongxin Jian

**Affiliations:** 1 Xi’an Thermal Power Research Institute Co., Ltd., Xi’an 710049, China; liyong@tpri.com.cn (Y.L.);; 2 State Key Laboratory of Porous Metal Materials, Northwest Institute for Nonferrous Metal Research, Xi’an 710016, China; 3 School of Mechanical Engineering, Xi’an Jiaotong University, Xi’an 710049, China

**Keywords:** Y_2_O_3_, Inconel 625 coating, laser cladding, microstructure, high-temperature corrosion behaviors

## Abstract

Water-cooled wall tubes are susceptible to high-temperature corrosion during service. Applying high-performance coatings via laser cladding on the tube surfaces can significantly enhance corrosion resistance and extend the service life of the tubes, providing substantial economic advantages. This paper prepared Y_2_O_3_/IN625 composite coating by means of high-speed laser cladding. Furthermore, the effects of Y_2_O_3_ addition on the microstructure evolution, hardness, as well as the high-temperature corrosion behaviors have been systematically investigated. The results show that Y_2_O_3_ addition can effectively refine the microstructure of the Inconel 625 coating, but the phase composition has little change. The coating’s hardness can also be improved by about 7.7%, reaching about 300 HV. Compared to Inconel 625 coating, the Y_2_O_3_-added composited coating shows superior high-temperature corrosion resistance, with the corrosion mass gain decreased by about 36.6%. The denser and tightly bonded Cr-rich oxides layer can be formed adjacent to the coating surface, which plays a predominant role in improving the coating corrosion resistance.

## 1. Introduction

In coal-burning electric power plants, waterwall corrosion is the predominant failure form inside the high-temperature boilers [1]. Due to the exposure to high-temperature combustion gases, including H_2_S, SO_2_, CO_2_, etc., waterwall tubes made by carbon steels degrade rapidly, and this can even result in pipe explosion [2,3,4]. This kind of accident contributes to irregular downtime in power plants, which causes significant economic losses. To address the problem, the direct method is to substitute the initial tube materials with high-performance materials with better corrosion resistance, which would undoubtedly increase the production cost. On the other hand, introducing protective coatings with superior corrosion resistance on the tubes’ surface can effectively decrease the corrosion failure of the boiler tube [5,6,7,8].

In the past years, plenty of studies have been conducted to develop high-performance coatings with superior corrosion resistance on waterwall steel [5,7,8,9]. Singh et al. [5]. investigated the hot corrosion behavior of HOVF-sprayed NiCrAlY/carbide composite coatings at 900 °C in a Na_2_SO_4_-60 wt.%V_2_O_5_ molten-salt environment. The composite coatings showed good protective performance due to the formation of SiO_2_ and the oxides of Ni, Al, and Cr. Vairs et al. [10] evaluated the corrosion behaviors of several NiCr and FeCr thermally sprayed coatings under a KCl-K_2_SO_4_ mixed salt environment. The results revealed that the Ni49Cr, Ni21Cr, and Fe30Cr coatings showed promising corrosion-resistant performance at 600 °C. Liu et al. [11] compared the hot corrosion performance of HVOF NiCrAlY and NiCrBSi coatings in a KCl environment at 700 °C and found that NiCrBSi showed superior corrosion resistance to NiCrAlY due to the denser structure and generation of a SiO_2_ protective layer. Until now, most of the current studies have focused on thermal-sprayed coatings in the application of boiler tube protection. According to the review by Singh et al. [12], the Ni-Cr based coatings can be utilized to combat hot corrosion effectively. However, the thermal-sprayed coatings have a few inherent disadvantages as high-temperature corrosion-resistant coatings. On the one hand, porosity is hardly avoided inside the coating, which may act as a channel for corrosive gas. On the other hand, the bonding strength between the coating and the substrate is usually weak, which may bring about spalling during the working process.

Compared with thermal-sprayed coatings, laser cladding can prepare dense coatings with metallurgical bonding to the substrate, and the cladded coatings possess low dilution rates, small heat-affected zones, and refined microstructures [13,14,15]. In this case, laser cladding is regarded as a potential technology to combat the corrosion to the boiler surface. Nevertheless, there are only a few investigations focusing on high-temperature corrosion in the boiler waterwall environment of laser cladding coatings. Gong et al. [16] investigated the corrosion properties of Ni-based alloy cladded coatings in the high-temperature chloride environment and proved the superior corrosion resistance to traditional stainless steel. Likewise, Cheng et al. [17] prepared FeCrAl laser cladding coatings and tested the high-temperature corrosion behaviors in mixed salts at 650 °C. The results showed that the Fe-13Cr-7Al coatings exhibited the best high-temperature corrosion resistance, achieving 73% of the Inconel 625 alloy coating. 

Among the laser cladding coating materials, the Inconel 625 alloy showed better comprehensive properties, including a good deposition property and excellent corrosion resistance [18,19,20,21,22,23,24,25,26,27,28]. There is potential to prepare an Inconel 625 alloy coating on the waterwall surface to ameliorate the corrosion problem. In this case, it is important to reveal the corrosion behaviors of Inconel 625 coatings in the environment of high-temperature combustion gases. On the other hand, the boiler waterwall has to suffer more severe corrosion with the development of the boiler device and combustion technology (such as low NOx burning and inferior coal burning). Thus, it is significant to further improve the high-temperature corrosion resistance of commercial Inconel 625 coatings. According to the literature [29,30,31], the addition of Y_2_O_3_ particles can facilitate a reduction in the porosity and crack initiation of the laser cladding coating. In addition, the coating’s microstructure can be refined to some degree by adding appropriate Y_2_O_3_, which is beneficial for modifying the mechanical and corrosion resistance. In this context, it is a promising method to further improve the high-temperature corrosion resistance of Inconel 625 coatings. However, there are no public reports concerning the high-temperature corrosion behaviors of laser cladding Inconel 625 coatings with Y_2_O_3_ addition. 

In this case, this work attempted to prepare a Y_2_O_3_-added Inconel 625 coating by means of high-speed laser cladding. Furthermore, the high-temperature corrosion properties have been evaluated and the intrinsic mechanisms have been systematically investigated. The obtained results are expected to provide guidelines for the design and preparation of high-quality protective coatings on boiler waterwalls. 

## 2. Materials and Methods

### 2.1. Coating Preparation

**Figure 1:**
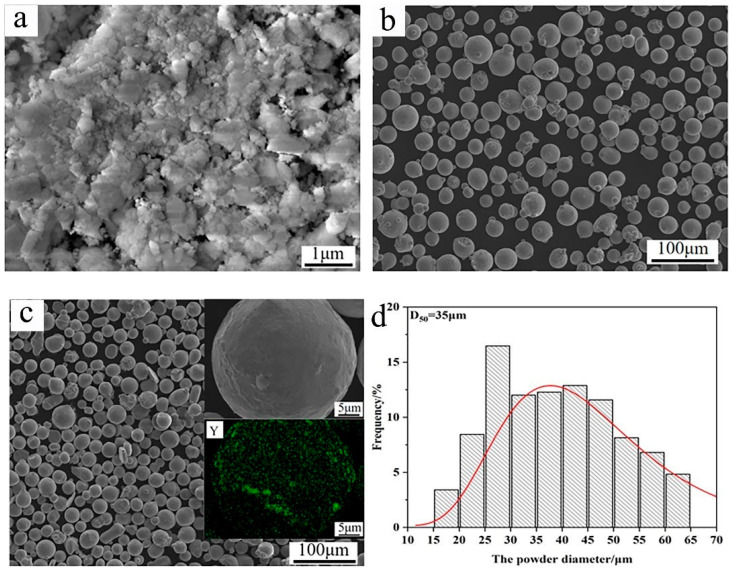
Raw materials for the preparation of high-speed laser cladding: (**a**) Y_2_O_3_; (**b**) Inconel 625; (**c**) mixed powders (Y representing for Y element); (**d**) size distribution diagram of the mixed powders.

In this work, a TP347 stainless steel pipe (Jiangsu Shagang Group Co., Ltd., Suzhou, China) was chosen as the substrate, with an average hardness of 165 HV. The chemical composition of TP347 is listed in Table 1. In addition, nano-Y_2_O_3_ and Inconel 625 powders (Xi’an Sailong Metal Materials Co., Ltd., Xi’an, China) were used as the raw materials, as shown in Figure 1a,b. Nano-Y_2_O_3_ powder exhibits agglomeration, while Inconel 625 powders display good spherical morphology. To obtain the raw powders of the composite coating, 0.3 wt.% Y_2_O_3_ was added into Inconel 625 powder, then evenly mixed by the method of mechanical ball milling. The weighed raw powders were mixed in a stainless steel milling jar and then milled on a planetary ball milling machine. The milling speed was 100 r/min and the lasting time was 3 h. After milling, the Y_2_O_3_ powder was evenly distributed on the surface of Inconel 625 powders, as shown in Figure 1c. The mixed powders still retained their spheroid morphology, and the powder diameter ranged from 15 to 65 μm, with an average size of 35, as seen in Figure 1. The measuring error of the average size was limited within 1%.

**Figure 2:**
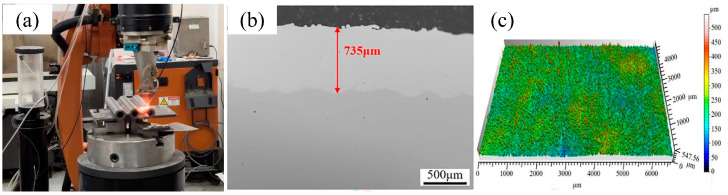
The laser cladding site and coating morphology of the Y_2_O_3_/Inconel 625 composite coating: (**a**) photo of the laser cladding site; (**b**) cross-section; (**c**) coating surface.

High-speed laser cladding experiments were conducted on a high-speed laser cladding system (ZKZM-4000, Zhongke Zhongmei Laser Technology Co., Ltd., Xi’an, China), which comprised a 4 kW fiber laser, a cladding head with synchronous powder feeding, a powder feeding machine, and a KUKA operating robot [13,14]. The wavelength of the laser was 1080 nm, and the size of the laser beam was 2 mm. Table 2 gives the main parameters for the laser cladding process. The cladding parameters herein were optimized in advance to guarantee the coating’s quality. Figure 2 shows the cross-section and surface morphologies of the prepared laser cladding Y_2_O_3_/Inconel 625 composite coating. The coating’s thickness was about 735 μm, and no apparent defects could be observed in the interface region. Simultaneously, the coating’s surface was relatively smoother, which can be attributed to the higher overlapping rate.

### 2.2. Microstructure Characterization

All the specimens for microstructure characterization and mechanical properties tests were picked up from the cladded pipe using the method of electric sparking cutting. Before microstructure observation, the sample surface was ground on SiC abrasive papers with different meshes and then polished on canvas with the addition of diamond suspension in order to eliminate the scratches. To reveal the grain morphology, the polished surface was etched with a solution of FeCl_3_ and ethyl alcohol. 

A scanning electron microscope (Hitachi SU3500, SEM, Hitachi, Tokyo, Japan) equipped with energy-disperse spectroscopy (EDS, Oxford Instruments, Abingdon, UK) was used to check the microstructure and the element composition of the laser cladding coating. The grain morphology was observed in the secondary electronic imaging mode with an acceleration voltage of 15 KV. Electron backscatter diffraction (EBSD, Oxford Instruments, Abingdon, UK) was also used to detect the crystal grains’ morphology and orientation characteristics with a step size of 1 μm. X-ray diffraction (XRD) measurements were performed using a Bruker D8 ADVANCE (Bruker Ltd., Ettlingen, Germany) with a Cu–K α source (λ = 1.5406 Å) scanning from 20° to 100° at a scan speed of 0.03°/s. 

### 2.3. Hardness Testing

The microhardness was measured from the coating surface to the coating/substrate interface along the building direction by using an HV100-TPTA Vickers hardness tester (Weiyee, Laizhou, China). The testing load was set at 100 g and the dwell time was 15 s. To guarantee the data’s repeatability, at least five tests were performed to obtain the average value. 

### 2.4. High-Temperature Sulfur-Containing Gas/Molten Corrosion Test

**Figure 3:**
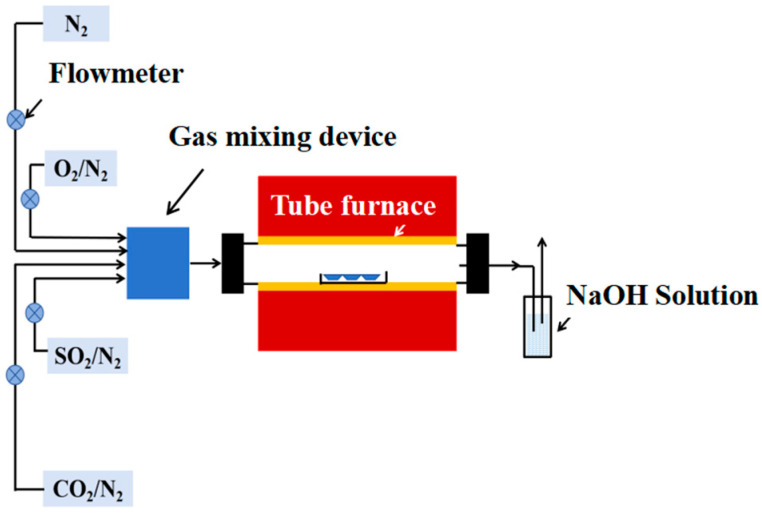
Schematic diagram of the high-temperature corrosion testing platform.

To simulate the work environment of a waterwall, a high-temperature corrosion test platform was established in this work (as shown in Figure 3), which consisted of a gas generation system, a heating chamber, and tail gas treating equipment. The gas composition was determined according to the boiler field data, which are shown in Table 3. Except for N_2_, O_2_, and CO_2_, 0.25 vol.% SO_2_ also existed in the combustion gas, which would cause severe corrosion of the waterwall surface. Considering the composite sulfate deposited on the waterwall surface, this work also introduced Na_2_SO_4_ and K_2_SO_4_ mixed salt onto the surfaces of tested specimens. To accelerate the corrosion rate, the temperature for the corrosion test was set as 650 °C. The total corrosion time was 168 h, and the tested specimens were weighed every 24 h to obtain the corrosion weight gain. An electronic balance with an accuracy of 0.01 mg was used to weigh the specimens before and after the corrosion test. To guarantee the repeatability, 3 specimens were tested for each sample to acquire the average value. 

After corrosion test, the corrosion surface as well as the cross-section were observed by SEM to analyze the corrosion characters. Furthermore, EDS and XRD were used to help clarify the elemental information and phase composition of the surface corrosion layer, respectively.

## 3. Results and Discussion

### 3.1. Microstructure Analysis

**Figure 4:**
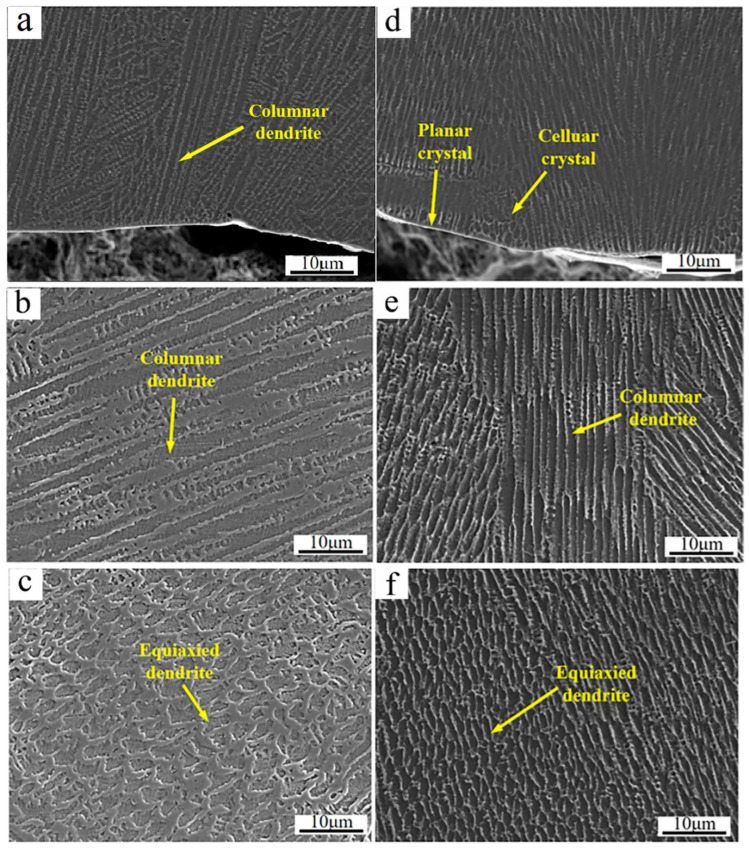
SEM micrographs of the coating’s cross-section: (**a**) bottom region of Inconel 625; (**b**) middle region of Inconel 625; (**c**) top region of Inconel 625; (**d**) bottom region of Y_2_O_3_/Inconel 625 composite coating; (**e**) middle region of Y_2_O_3_/Inconel 625 composite coating; (**f**) top region of Y_2_O_3_/Inconel 625 composite coating.

Figure 4 shows the SEM micrographs of the cross-section of the cladded Inconel 625 and Y_2_O_3_/Inconel 625 composite coatings. As shown, the high-speed laser cladding coatings were mainly composed of dendrites and the inter-dendritic structure. This agrees well with the previous studies that have stated that γ-Ni dendrites dominate the microstructure of Ni-based cladding coatings [26,27,32,33]. And the morphology of the dendrite varied in different regions of the cladded coatings. In the bottom region, the dendrites mainly showed features of cellular crystals and columnar crystals. Differently from some high-speed laser cladding coatings reported before [34], no apparent planar crystals could be observed except for a very thin layer, which was mainly attributed to the relatively slow scanning speed. In the middle region, the dendrites were dominated by columnar crystals for both coatings. However, the dendrites were nearly equiaxed crystals on the top region, which originated from the relatively higher cooling rate [14]. Considering the same cladding parameters, the microstructure features in different regions were similar for the cladded Inconel 625 and Y_2_O_3_/Inconel 625 composite coatings. However, it was visible that the microstructure could be obviously refined after the addition of Y_2_O_3_ particles. This phenomenon was also found in the laser cladding Inconel 718 coating [29], in which the average grain size was reduced by 39.5%. In summary, it can be concluded that the addition of Y_2_O_3_ is beneficial for refining the microstructure of the Inconel 625 laser cladding coating.

**Figure 5:**
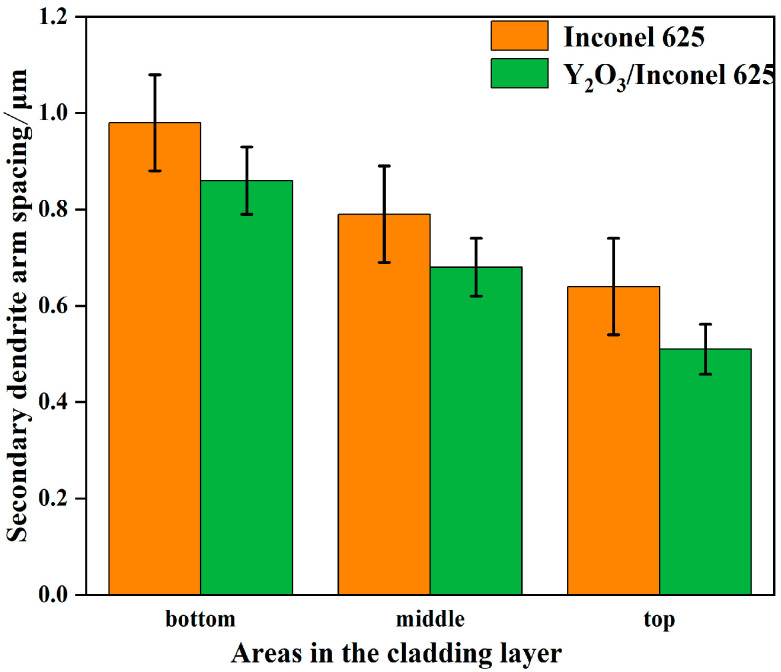
Secondary dendritic arm spacing of Y_2_O_3_/Inconel 625 composite coating.

**Figure 6:**
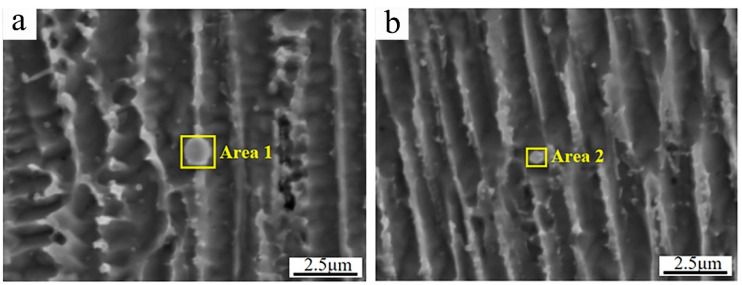
Magnified SEM micrographs of high-speed laser cladding Y_2_O_3_/Inconel 625 coating: (**a**) inter-dendritic region; (**b**) inside dendrite.

Figure 5 shows the secondary dendrite arm spacings of γ-Ni measured in the cladded Inconel 625 and Y_2_O_3_/Inconel 625 composite coatings. Firstly, it can be found that the secondary dendrite arm spacing gradually decreased from the bottom region of the coating to the top region, which is mainly attributed to the difference in cooling rate during the cladding process [13,35]. During the cladding process, the cooling rate was the highest in the top region of the melt pool, while it was the lowest in the bottom region. To further clarify the effect of Y_2_O_3_ addition on the microstructure, Figure 6 displays the magnified SEM micrographs of the Y_2_O_3_/Inconel 625 composite coating. It can be found that the added Y_2_O_3_ particles were distributed randomly inside the coatings, which could be confirmed based on the EDS results in Table 4. The particles could not only be found in the inter-dendritic regions, but also inside the dendrite. During the solidification process, the Y_2_O_3_ particles acted as heterogeneous nucleation sites, which facilitated the nucleation of γ-Ni grains. In this case, the microstructure could be refined, with more grains growing competitively.

**Figure 7:**
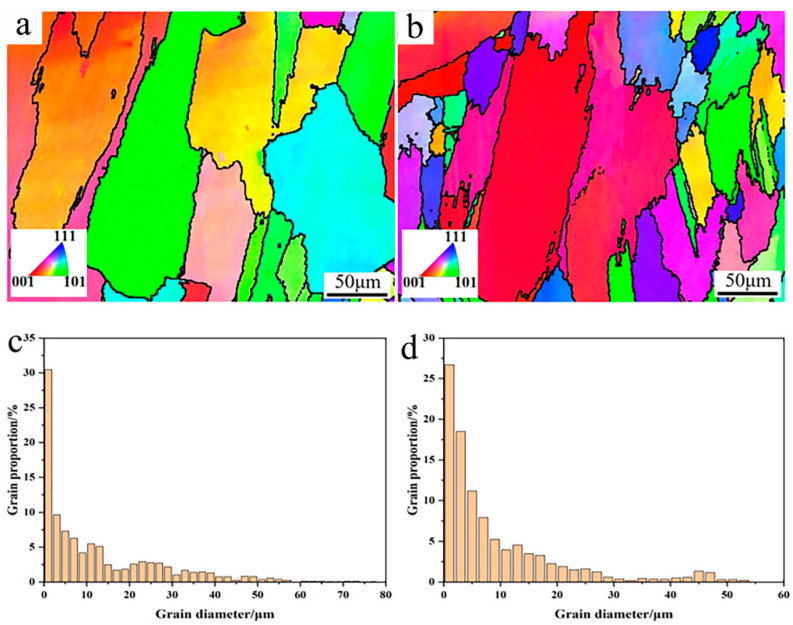
EBSD analysis of the cladded Y_2_O_3_/Inconel 625 composite coating: (**a**) IPF of Inconel 625 coating; (**b**) IPF of Y_2_O_3_/Inconel 625 composite coating; (**c**) grain size distribution of Inconel 625 coating; (**d**) grain size distribution of Y_2_O_3_/Inconel 625 composite coating.

To further confirm the microstructure evolution, EBSD analysis was conducted to analyze the grain size and crystal orientation, as shown in Figure 7. From the IPF figures in Figure 7a,b, it can be found that the γ-Ni crystal grains mainly showed columnar grains to some degree, which is mainly attributed to the heat flow during the cooling process [14]. After Y_2_O_3_ addition, more small grains could be observed, which agrees well with the SEM microstructure observation above. According to the distribution proportion of the grain size, more small grains ranging from 10 to 20 μm appeared in the Y_2_O_3_/Inconel 625 coating, which confirms the fact that the composite coating possessed a finer microstructure.

**Figure 8:**
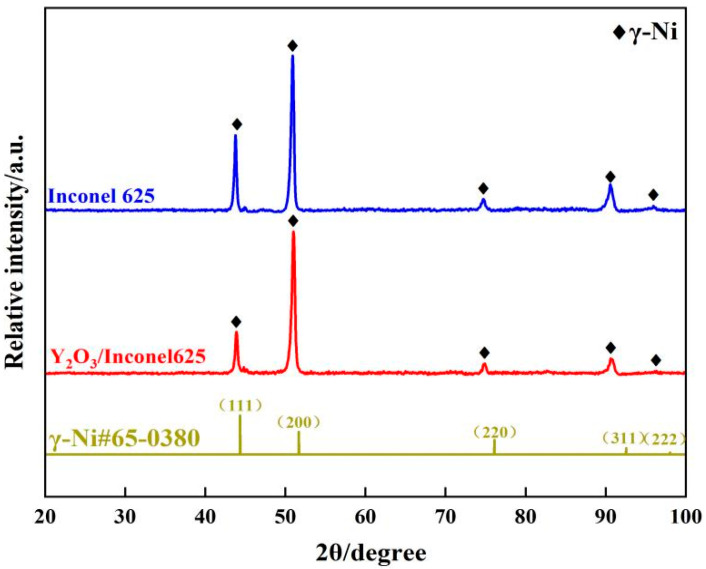
XRD patterns of high-speed laser cladding Y_2_O_3_/Inconel 625 coating (γ-Ni#65-0380 representing for the standard card of X-rays diffraction).

To ensure the phase composition, Figure 8 gives the XRD patterns of the laser cladding Inconel 625 and Y_2_O_3_/Inconel 625 composite coatings. As shown, the cladded coatings were mainly composed of γ-Ni phase. The inter-dendritic structures can hardly be detected because the content is too small. Likewise, it is difficult to find the diffraction peaks of the added Y_2_O_3_. By comparison, the diffraction peaks did not change after the addition of Y_2_O_3_, but the intensity varied greatly. It can be found that the (1 1 1) diffraction peak seemed to decrease slightly, which implies that the crystal grain orientation may have shown some change. In summary, the addition of Y_2_O_3_ had little influence on the phase composition of the laser cladding Inconel 625 coating.

### 3.2. Hardness

**Figure 9:**
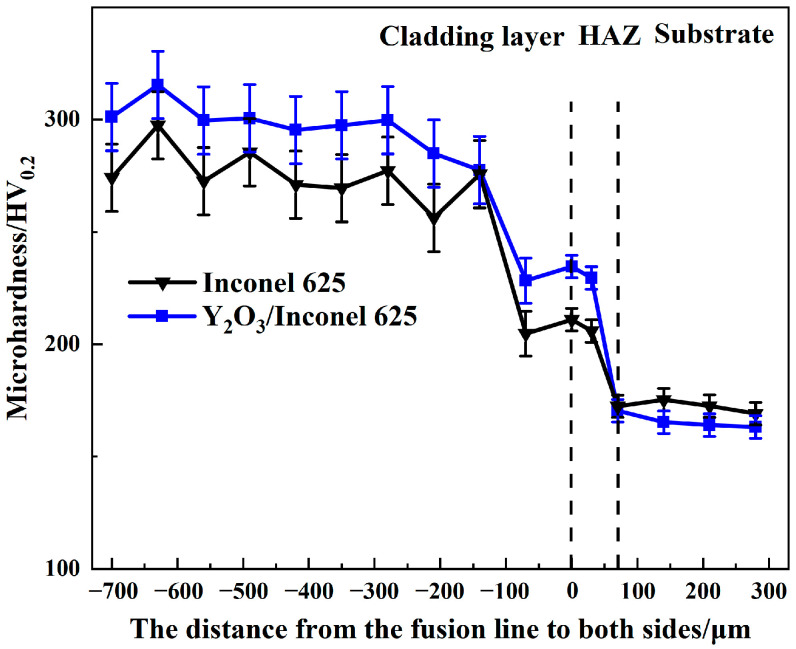
Cross-section hardness of the high-speed laser cladding Y_2_O_3_/Inconel 625 coating.

Hardness is an important factor affecting the wear resistance of cladded coatings. Coatings with higher hardness can better resist the erosion wear from the flying ashes in the boiler [36]. The Vickers hardness was measured along the cross-sections of two laser cladded coatings, as shown in Figure 9. By comparison, both the cladded coatings had higher hardness values than the substrate of TP347. And the Y_2_O_3_/Inconel 625 composite coating showed a slightly higher hardness than the Inconel 625 coating, reaching about 300 HV. Based on the microstructure analysis above, the Y_2_O_3_/Inconel 625 composite coating possessed a finer microstructure, which was beneficial to improving the hardness [37]. Furthermore, the existence of small-sized Y_2_O_3_ particles may have also acted as the secondary phase, which could also have enhance the hardness. Therefore, the higher hardness of the composite coating is reasonable considering the difference in microstructure [38].

### 3.3. High-Temperature Corrosion Behaviors in Sulfur-Containing Gas/Molten Salts

**Figure 10:**
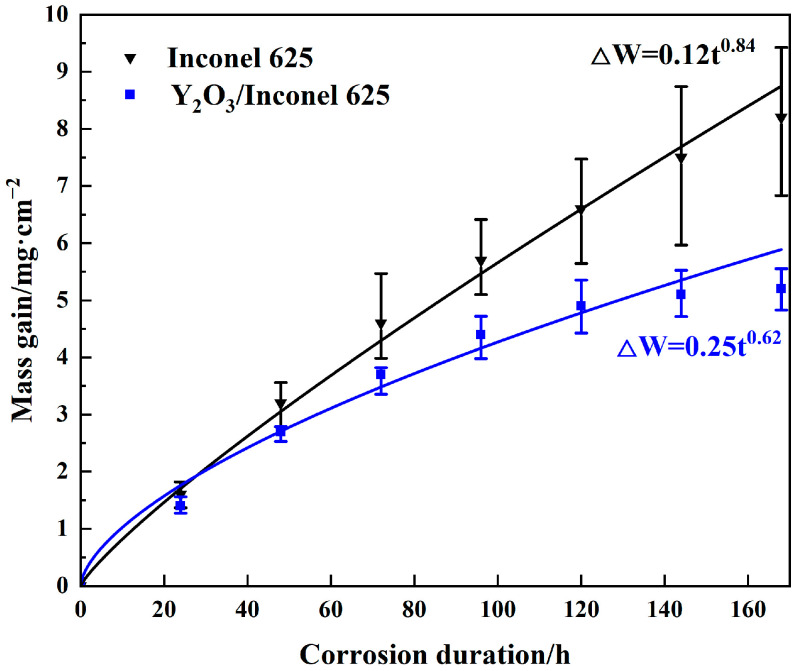
Thermo-gravimetric curves of the high-speed laser cladding Y_2_O_3_/Inconel 625 coating.

Figure 10 shows the thermo-gravimetric curves of the two cladded coatings under the sulfur-containing gases and mixed salts at 650 °C. As shown, the corrosion mass gains of both coatings showed exponentially increasing trends with the corrosion time. After 168 h of corrosion, the mass gain of Y_2_O_3_/Inconel 625 composite coating was obviously lower than that of the Inconel 625 coating; it decreased by about 36.6%. This result indicates that Y_2_O_3_ addition can greatly improve the high-temperature corrosion resistance of the laser cladding Inconel 625 coating. According to the curve-fitting result, it can be found that the mass gains showed the exponential growth law, but not the parabolic growth law. And the exponential growth of the Y_2_O_3_/Inconel 625 composite coating was 0.62, which is 26.2% lower than that of the Inconel 625 coating. Thus, it can be concluded that the laser cladding Y_2_O_3_/Inconel 625 composite coating showed superior corrosion resistance to the initial Inconel 625 coating.

**Figure 11:**
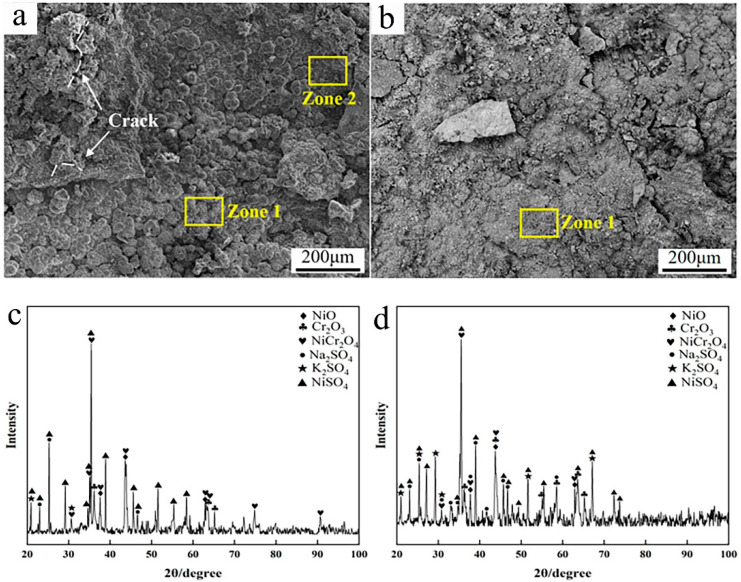
SEM micrographs and XRD patterns of the corrosion surface after 168 h: (**a**) micrographs of cladded Inconel 625 coating; (**b**) micrographs of Y_2_O_3_/Inconel 625 composite coating; (**c**) XRD of cladded Inconel 625 coating; (**d**) XRD of Y_2_O_3_/Inconel 625 composite coating.

To further elucidate the corrosion behaviors of the two coatings, the corrosion surface morphology and phase composition were analyzed. As shown in Figure 11a,b, a great deal of corrosion products could be found to cover the specimen’s surface after 168 h corrosion. As for the Inconel 625 coating, the corrosion surface showed typical morphologies of large bulks and bulge-like peaks. Based on the EDS analysis, these bulges were rich in Ni, S, and O, indicating the surface corrosion products to be sulphates containing Ni. From the XRD patterns in Figure 11c, a great deal of nickel sulfates and oxides of Ni and Cr could be detected, except for the initial sulfates’ mixed salts. This further confirms the surface corrosion products to be oxides and sulfates of Ni and Cr. In addition, it was found that a few cracks arose on the surface of the corrosion products, which is supposed to have been caused by the stress during the cooling process. According to the previous studies [36,39], the corrosion or oxidation product layers tend to crack when the thickness is greater.

As for the Y_2_O_3_/Inconel 625 composite coating, the corrosion surface was quite different. No apparent bulge-like products formed on the corrosion surface except for a few irregular bulks. And the corrosion surface seemed smoother, which implies more slight corrosion. Based on the EDS analysis, the chemical composition of the corrosion surface was similar for the two coatings. Likewise, the phase composition was also similar according to the XRD patterns. This result is easy to understand because the two coatings basically possessed the same chemical composition, except for the addition of tiny Y_2_O_3_. Correspondingly, the corrosion reactions were supposed to be the same during the high-temperature corrosion process. Regarding the differences in corrosion resistance and surface morphology, the coating’s microstructure and tiny added Y_2_O_3_ were deemed to play important roles [29].

**Figure 12:**
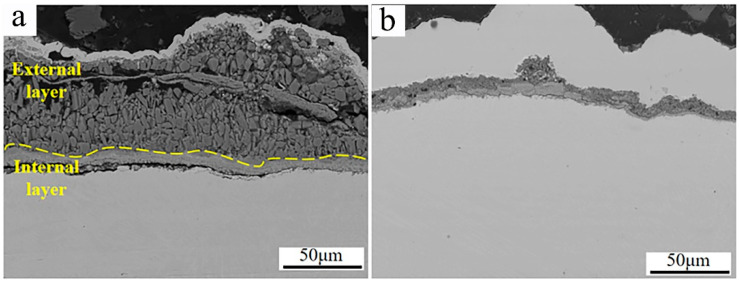
SEM micrographs of the cross-section morphology after 168 h of corrosion: (**a**) Inconel 625; (**b**) Y_2_O_3_/Inconel 625 composite coating.

Figure 12 shows the SEM micrographs of the cross-section morphologies of Inconel 625 and Y_2_O_3_/Inconel 625 composite coatings after 168 h of high-temperature corrosion. From Figure 12a, it can be found that a thick corrosion layer formed on the surface of the Inconel 625 coating. And the high-temperature corrosion products of the Inconel 625 coating can be divided into two distinctive layers. The internal layer was relatively compact and rich in Cr, according to the EDS analysis in Table 5, indicating it to be the oxide of chromium [37,40]. However, the external layer seemed to be loosened and filled with holes and cracks, comprising small blocks. Based on the EDS analysis, the external layer was mainly composed of Ni, O, and S, which is exactly consistent with the corrosion products on the surface. In general, the layer of dense and continuous corrosion products can hinder the corrosion reaction so that the corrosion resistance is improved [28,41]. But the loosened corrosion product layer cannot improve the corrosion resistance because it is difficult to impede the corrosive medium in order to invade it [42]. By comparison, only a thin corrosion product layer formed on the surface of the Y_2_O_3_/Inconel 625 composite coating. This also confirms its superior corrosion resistance under the sulfur-containing gases and mixed salts at 650 °C. By the way, despite the fact that the corrosion product layer was not very compact for the Inconel 625 coatings, a dense and continuous Cr-rich oxide layer formed adjacent to the sample surface. This accounts for the fairly good high-temperature corrosion resistance of the Inconel 625 coating.

**Figure 13:**
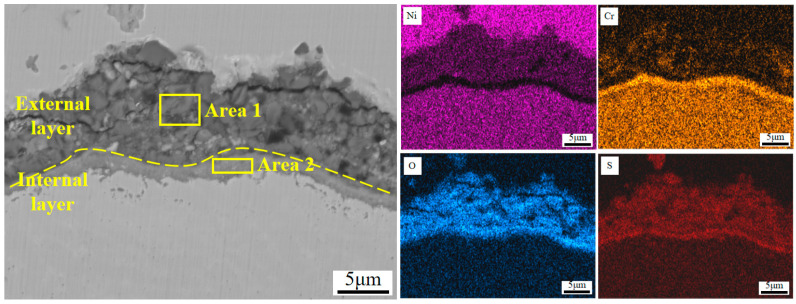
Magnified SEM micrographs of the cross-section morphology of Y_2_O_3_/IN625 composite coating.

To further clarify the corrosion behaviors of the Y_2_O_3_/Inconel 625 composite coating, the magnified SEM micrographs of the cross-section are displayed in Figure 13. From the magnified micrographs, it can be found that the corrosion product layer could also be divided into two layers, which is similar to that of Inconel 625 coating to some degree. However, the external layer was obviously thinner and more compact for the Y_2_O_3_/Inconel 625 composite coating. This may be attributed to the refined microstructure of the Y_2_O_3_-added Inconel 625 coating [43]. On the other hand, the internal layer of the corrosion products was also detected to contain the oxides of chromium. The Cr-rich oxide layer was visibly dense and tightly bonded to the coating surface. Compared to the internal corrosion layer of Inconel 625 coating, the tightly bonded Cr-rich oxides were expected to have a better protective effect. Based on the EDS result in Table 6, the Y element could be detected inside the internal Cr-rich oxide layer, indicating that the addition of Y_2_O_3_ is beneficial to facilitate densification and strong interface bonding [42]. The intrinsic mechanism will be further investigated in the future. Therefore, it can be concluded that the addition of Y_2_O_3_ can facilitate the formation of a dense and strongly bonded internal Cr-rich oxide layer, which can effectively hinder the interdiffusion process of the metal atoms and corrosive matters. Correspondingly, the growth rate of the sulphates of nickel can be greatly decreased, accounting for the relatively thinner external layer. In summary, the addition of Y_2_O_3_ can not only modify the microstructure of the Inconel 625 coating, but also improve the high-temperature corrosion resistance.

## 4. Conclusions

To obtain laser cladding coatings with superior corrosion resistance, the authors proposed to add tiny Y_2_O_3_ into an IN625 coating, which effectively improved the hardness and high-temperature corrosion resistance under a sulfur-containing gases and mixed salts environment. Simultaneously, the intrinsic mechanism of the high-temperature corrosion was also clarified by analyzing the corrosion product layer. The specific conclusions were drawn as below:

1. The high-speed laser cladding Y_2_O_3_/IN625 composite coating is mainly composed of γ-Ni dendrites and an inter-dendritic structure; the addition of Y_2_O_3_ can effectively refine the microstructure, but not change the phase composition.
2. The hardness of the Y_2_O_3_/IN625 composite coating can be improved by about 7.7% compared to the Inconel 625 coating, which is mainly attributed to the refined microstructure and strengthening effect of the Y_2_O_3_ particles.
3. The laser cladding Y_2_O_3_/IN625 composite coating shows superior high-temperature corrosion resistance, with the mass gain decreased by about 36.6%. Y_2_O_3_ particles enhance the densification of the internal Cr-rich oxide layer and improve the interface bonding strength to the sample surface.

## Figures and Tables

**Table 1:** Chemical composition of TP347H stainless steel/wt.%.

Cr	Ni	Mn	Si	Nb	C	Fe
17.39	9.08	1.29	0.49	0.48	0.043	Bal.

**Table 2:** Laser cladding parameters of Y_2_O_3_/Inconel 625 composite coating.

Laser Power/W	Scanning Speed/mm·min^−1^	Powder Feeding Rate /g·s^−1^	Feeding Powder Gas /L·min^−1^	Overlapping Rate /%
3000	3000	0.30	5	80

**Table 3:** Composition of the corrosion gas/vol.%.

Species	N_2_	CO_2_	O_2_	SO_2_
Ratio	81.25	14.9	3.6	0.25

**Table 4:** EDS analysis result of high-speed laser cladding Y_2_O_3_/Inconel 625 coating/wt.%.

Regions	Ni	Cr	Mo	Nb	Fe	Al	Y	O
Area 1	36.25	19.50	5.62	7.18	6.77	9.08	3.79	11.81
Area 2	40.12	18.04	6.32	4.94	6.73	9.79	2.36	11.70

**Table 5:** EDS elemental analysis of the corrosion surface of cladded Inconel 625 coating/at.%.

Elements	Ni	Cr	Mo	Nb	Fe	Al	O	S
External layer	23.39	0.39	/	/	0.60	/	60.94	14.68
Internal layer	4.72	29.48	5.12	1.86	1.05	0.32	47.72	9.73

**Table 6:** EDS elemental analysis of the corrosion product layer of Y_2_O_3_/Inconel 625 composite coating/at.%.

Elements	Ni	Cr	Mo	Nb	Fe	Al	Y	O	S
Area 1	19.83	0.24	-	-	0.16	0.13	0	60.84	18.80
Area 2	1.96	25.97	4.59	1.87	0.17	0.33	0.33	52.49	12.29

## Data Availability

The original contributions presented in the study are included in the article, further inquiries can be directed to the corresponding author.
